# Social stress and glucocorticoids alter PERIOD2 rhythmicity in the liver, but not in the suprachiasmatic nucleus

**DOI:** 10.1016/j.yhbeh.2020.104683

**Published:** 2020-04

**Authors:** S.M. Ota, R.A. Hut, S.J. Riede, P. Crosby, D. Suchecki, P. Meerlo

**Affiliations:** a Groningen Institute for Evolutionary Life Sciences, University of Groningen, the Netherlands; b Department of Psychobiology, Universidade Federal de São Paulo, São Paulo, Brazil; c MRC Laboratory of Molecular Biology, Francis Crick Ave, Cambridge, United Kingdom

**Keywords:** Social stress, Glucocorticoids, Suprachiasmatic nucleus, Peripheral oscillator, Clock gene expression, Circadian disruption

## Abstract

Circadian (~24 h) rhythms in behavior and physiological functions are under control of an endogenous circadian pacemaker in the suprachiasmatic nucleus (SCN) of the hypothalamus. The SCN directly drives some of these rhythms or serves as a coordinator of peripheral oscillators residing in other tissues and organs. Disruption of the circadian organization may contribute to disease, including stress-related disorders. Previous research indicates that the master clock in the SCN is resistant to stress, although it is unclear whether stress affects rhythmicity in other tissues, possibly mediated by glucocorticoids, released in stressful situations. In the present study, we examined the effect of uncontrollable social defeat stress and glucocorticoid hormones on the central and peripheral clocks, respectively in the SCN and liver. Transgenic PERIOD2::LUCIFERASE knock-in mice were used to assess the rhythm of the clock protein PERIOD2 (PER2) in SCN slices and liver tissue collected after 10 consecutive days of social defeat stress. The rhythmicity of PER2 expression in the SCN was not affected by stress exposure, whereas in the liver the expression showed a delayed phase in defeated compared to non-defeated control mice. In a second experiment, brain slices and liver samples were collected from transgenic mice and exposed to different doses of corticosterone. Corticosterone did not affect PER2 rhythm of the SCN samples, but caused a phase shift in PER2 expression in liver samples. This study confirms earlier findings that the SCN is resistant to stress and shows that clocks in the liver are affected by social stress, which might be due to the direct influence of glucocorticoids released from the adrenal gland.

## Highlights

• Chronic social defeat does not affect phase or period of the circadian rhythm.
• Chronic social defeat does not alter PER2 expression in the suprachiasmatic nucleus.
• Chronic social defeat phase shifts PER2 expression in the liver.
• PER2 expression in the liver is altered by corticosterone *in vitro*.

## 1 Introduction

Circadian (~24 h) rhythms in physiology and behavior are the result of interacting endogenous rhythms that reside in most, if not every cell in the body (Dibner et al., 2010; Leise et al., 2012, Saper, 2013; Yoo et al., 2004). These rhythms persist even in isolated tissues and in the absence of environmental cycles that could drive them, *e.g.* in liver, lung, kidney and pituitary (Yamazaki et al., 2000; Yoo et al., 2004). The endogenous circadian rhythms generated in different tissues and organs are coordinated by a master clock located in the hypothalamic suprachiasmatic nucleus (SCN). The SCN not only coordinates other internal rhythms with each other, but also synchronizes them to the external environment. The SCN receives direct light input from the retina, which can reset the master clock, maintaining the endogenous clock system in pace with the environmental light-dark cycle (Dibner et al., 2010; Saper, 2013; Rosenwasser and Turek, 2015). The circadian system thus provides a precise internal time-of-day-representation that aids optimal timing of physiological processes and behavior in rhythmic environments (Dibner et al., 2010).

It is generally thought that disturbance of the circadian system and disruption of the normal coordination between internal rhythms can have a negative impact on performance, well-being and health. In the long run, circadian dysfunction may contribute to the development of diseases, including the pathogenesis of stress-related disorders (Healy, 1987; Schnell et al., 2014). Stress is often believed to be an intervening factor on circadian function and, eventually, any resulting change in circadian organization might, in turn, contribute to the development of stress disorders (for review, see Koch et al., 2017). This understanding is partly based on studies showing that stress-related disorders are often associated with changes in some aspects of rhythmicity, such as disturbance of the sleep-wake rhythm, altered temperature profile and changes in the daily pattern of hormone release (for review, see Meerlo et al., 2002). However, whether such changes in overt rhythms are truly caused by a disturbance of the endogenous circadian timing system remains uncertain.

In fact, much of the available data suggest that the master clock in the SCN is highly resistant to the effects of stress and stress hormones (Richter, 1967; Meerlo et al., 2002). For example, studies with restraint stress (Tahara et al., 2015) and unpredictable chronic mild stress (Logan et al., 2015) have demonstrated that these types of stress do not affect phase or period of the clock protein PERIOD2 (PER2) expression in the SCN. Even severe social defeat stress, which leads to long-lasting changes in physiology and behavior (Koolhaas et al., 1997), does not seem to affect the SCN (Meerlo et al., 2002). It was shown that acute social defeat stress lead to severe disturbances in the amplitude and shape of daily rhythms of activity, body temperature and heart rate, without affecting the endogenous phase and period of these rhythms under constant conditions (Meerlo et al., 1997, Meerlo and Daan, 1998). Moreover, our recent study in mice show that even repeated defeat for 10 consecutive days, although leading to a strong suppression of overall activity, does not affect the endogenous period and phase of the activity rhythm (Ota et al., 2018). However, while the SCN may not be sensitive to disruption by stress, it is still unclear whether stress affects clocks and rhythms in other tissues, which might then lead to a state of internal desynchronization with potential detrimental health effects.

One possible mechanism by which stress may directly affect peripheral clocks while leaving the master clock in the SCN unaffected is the release of glucocorticoid hormones. Importantly in this context, glucocorticoid receptors (GRs) are abundantly present in many tissues and organs, including the liver, but not the adult SCN (Rosenfeld et al., 1988). Moreover, the expression of some clock genes can be modulated by glucocorticoids, by binding of the GR to a glucocorticoid response element (GRE) in the promoter region of these genes (Segall and Amir, 2010). Administration of a synthetic glucocorticoid was found to shift the phase of clock gene transcription rhythms in peripheral organs, such as the liver, kidney and hearth (Balsalobre et al., 2000).

The aims of the present study were to determine whether chronic social stress would affect the SCN and/or the peripheral liver clock, and if that was the case, whether the glucocorticoid hormone corticosterone played a role on the stress effects. In the first experiment, adult male mice were subjected to repeated social defeat stress for 10 consecutive days, followed by *ex vivo* assessment of the PER2 rhythm in isolated liver and SCN samples. In the second experiment, we tested the direct effects of glucocorticoid stress hormone on isolated liver and SCN samples by adding corticosterone (CORT) to the culture medium, again followed by assessment of the PER2 rhythm. In both experiments we used transgenic PERIOD2::LUCIFERASE (PER2::LUC) mice, which produce a PER2::LUC fusion protein that allows for prolonged and continuous tracking of PER2 expression by means of measurement of luciferase-driven bioluminescence (Yoo et al., 2004).

## 2 Material and methods

### 2.1 Experiment 1. Effects of social defeat stress on running-wheel activity and PER2 rhythms

#### 2.1.1 Animals and housing

Twenty male PER2::LUC knock-in mice with a C57BL/6 background from our own colony were used as experimental animals and assigned to either a control or a social defeat group. The animals were individually housed in cages with a running wheel. Ten male CD-1 mice (from Charles River, Sulzfeld, Germany) were used as aggressors for the social defeats. The CD-1 mice were individually housed in a different room, where social defeats occurred. All mice had free access to food and water throughout the study and the rooms were temperature controlled (21 ± 1 °C). The experiments were conducted in accordance with the Dutch rules and regulations and approved by the Central Authority for Scientific Procedures on Animals (CCD).

#### 2.1.2 Experimental design

**Fig. 1:**
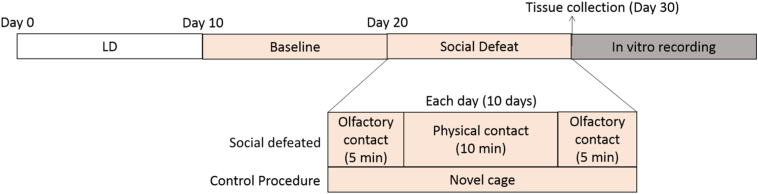
Timeline of experiment 1. The animals were habituated to a 12: 12 LD cycle for at least 10 days, after which they were transferred to a room with constant dim red light. For the next 10 days, the mice were undisturbed and baseline running wheel activity was recorded. Subsequently, the mice in the Social defeated group were submitted to the social stress once a day, for 10 days, as shown in the scheme, while the control animals were handled and placed in a different cage. After 10 days of social stress, the animals were euthanized and liver and SCN tissues were collected for the *in vitro* recording.

**Fig. 2:**
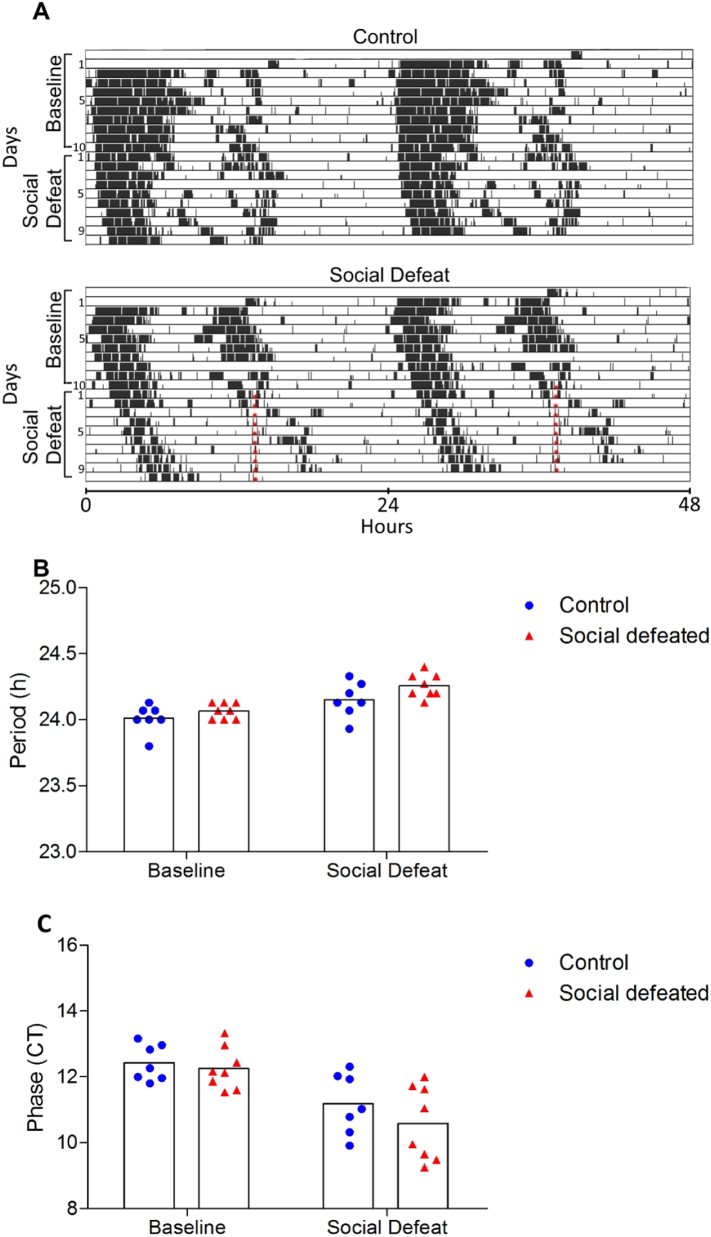
Panel A) Representative actograms of a control and a social defeated mouse. Red dots indicate when handling or social stress occurred. Observe activity suppression, especially in the end of active phase in the second actogram. Panel B) Period of activity rhythm during Baseline and Social Defeat days. Bars represent mean and symbols represent each individual animal. Panel C) Activity onset phase during Baseline and Social Defeat days. No difference between groups or time was observed. Bars represent mean and symbols represent each individual animal. (For interpretation of the references to color in this figure legend, the reader is referred to the web version of this article.)

The experimental mice were maintained under a 12:12 LD cycle until the start of the study, when they were transferred to constant dim red light. Running wheel activity was recorded and analyzed for two time-blocks, baseline and stress, each consisting of 10 days. During the 10-day stress phase, half of the mice were subjected to a daily social defeat. The other half served as control and were picked up and moved to a new cage for the same duration of time (see Fig. 1). The daily social defeat stress and control procedures took place at a fixed external time of day. On the day of the first defeat session, this was near the end of the active phase (around CT 23). As can be seen in Fig. 2, social defeat always took place at the same time of the day (*zeitgeber time* - ZT), but because animals were in free-running, this ZT might not have corresponded to the same internal time (*circadian time* - CT). Therefore, social stress was applied in a range of circadian phases (around the middle to the end of the active phase). One hour after the last defeat, mice were euthanized and SCN and liver tissues were collected for *in vitro* measurement of PER2 expression.

#### 2.1.3 Social defeat stress

Social defeat sessions took place under dim red light, similar to that in the home room of the experimental mice and care was taken to not expose them to any other light. Each social defeat session had a total duration of 20 min, divided in 3 phases (see Fig. 1). Phase 1 (5 min) was the initiation, during which the experimental animal was placed in the aggressor's cage, separated by a transparent and perforated acrylic wall, allowing olfactory and visual contact. Phase 2 (10 min) was the actual phase of physical interaction and defeat that started by removing the wall, after which the aggressor threatened and attacked the experimental animal. If during this phase, the intruder received >10 attacks before 10 min, the animals were separated and the remaining time was added to Phase 3. In Phase 3 (5 min), the mice were separated by the wall again. At the end of the procedure, experimental animals returned to their home cage. Social defeated animals were exposed to a new aggressor each day, to avoid habituation. Control mice were placed in an empty cage during the time the animals from the defeat group were exposed to social stress.

#### 2.1.4 Tissue preparation and *in vitro* recording

The procedures for tissue preparation and *in vitro* measurement of PER2 expression were similar to a previously described procedure, with minor adaptations (Yamazaki and Takahashi, 2005). Briefly, animals were euthanized by decapitation 1 h after the last defeat, still under red light. The eyes were also removed to fully exclude light reception by the retina. The remainder of the procedure was done in the light. Coronal brain sections (200 μm) were cut with a vibratome and placed in chilled Hanks' buffered salt solution (HBSS). Both SCN were later separated from the rest of the brain using a scalpel and placed in a dish with culture plate inserts and pre-warmed recording medium. A piece of the left lateral lobe of the liver was dissected and slices of approximately 1 mm were cut with a scalpel, also in chilled HBSS. Two liver samples from each animal were taken and placed in separate dishes with pre-warmed recording medium. The medium used in the present study was the same as published standards (Yamazaki and Takahashi, 2005), except that the B27 supplement was substituted by modified NS21 (Crosby et al., 2017) without CORT. The dishes with the samples were placed in the recorder and light emission, as reporter for PER2 expression, was measured for 4 days (LumiCycle, Actimetrics Inc., Evanston, IL).

#### 2.1.5 Data analysis

Running wheel activity was recorded in 2 min bins and analyzed with ChronoShop 1.04 (Spoelstra, 2015) for calculation of the period using the periodogram analysis based on the Sokolove and Bushell algorithm (Sokolove and Bushell, 1978). The daily onset phase of the activity rhythm was determined using a method similar to that described by Meerlo et al. (1997). Activity data was smoothened by a 1 h running average and the activity onset phase was defined as the time the 1 h smoothened data exceeded a 24 h running average. Afterwards, the times were transformed in circadian time (CT) for each animal, based on its period. Total activity per day as well as activity profile were also calculated in excel by aligning the activity counts according to the free-running period for each animal in each 10-day block. We tested the effects of stress on phase and period using repeated measures ANOVA, with between-subjects factor GROUP (Control and Social defeated) and within-subjects factor TIME (Baseline, Social defeat). Repeated measures ANOVA with between-subjects factor GROUP and within-subjects factor DAYS (1–10 day in each block) was used to test the effect on total activity per day. Analysis of the effects of stress on activity profile was done by repeated measures ANOVA with between-subjects factor GROUP and HOURS (24 circadian hours). Newman–Keuls test was used as a post-hoc when necessary. Results were considered statistical significant when p < 0.05.

Data analysis included hour 36 to 120 (hour 0 corresponded to start of bioluminescence recording). The first 24 h were excluded because the cellular bioluminescence during this time may exhibit changes related to dissection and media change. The analysis started at hour 36 due to the method used to remove drift in the average bioluminescence level. The drift in the bioluminescence recording trace was removed (detrended) using a 24 h moving average. The detrended data were then analyzed in GraphPad Prism (version 7.00 for Windows, GraphPad Software, La Jolla California USA) by fitting a cosine wave, accounting for damping of oscillatory amplitude, described by Crosby et al. (2017). The period of the fitted cosine was used as a measure for the period of the PER2 rhythm. The phase was determined by selecting the second peak of PER2::LUC rhythm in each sample. When both liver samples for a given animal survived, the results of these two samples were averaged. A Student's *t*-test was used to analyze the effects of social stress on period and phase of the PER2::LUC rhythm. For Student's *t-*tests, effect sizes were calculated using Cohen's d (Cohen, 1988; Lakens, 2013) (http://www.campbellcollaboration.org/escalc/html/EffectSizeCalculator-SMD1.php). An effect size of 0.20 is considered small, 0.50 is considered moderate and 0.8 is considered large. Eta squared (η^2^) and partial eta squared (η^2^_p_) were calculated for ANOVA and repeated measures ANOVA, respectively, using STATISTICA (TIBCO Software Inc. [2018]. Statistica [data analysis software system], version 13. http://tibco.com.) and a η^2^ = 0.01 is considered small, η^2^ = 0.06 is considered medium, and η^2^ = 0.14 is considered large.

### 2.2 Experiment 2. Direct effects of glucocorticoids on PER2 rhythm

#### 2.2.1 Animals and housing

Twenty male PER2::LUC knock-in mice with a C57BL/6 background from our own colony. The animals were maintained in a room with controlled temperature and 12:12 LD cycle, housed in groups of maximum 4 per cage.

#### 2.2.2 Experimental design

The mice in this experiment were maintained in a room with controlled temperature and 12:12 LD cycle, housed in groups of maximum 4 per cage. The animals remained undisturbed until the moment of tissue collection. Half of the animals was killed at ZT 11 and the other half at ZT 23. Liver samples and brain sections containing the SCN were directly exposed to CORT *in vitro*. Although we aimed to assess the effect of chronically elevated CORT levels, we collected tissues at 2 different time points to determine whether or not the starting time of the treatment in itself could have an effect.

#### 2.2.3 Tissue collection and processing

The procedures for tissue preparation and *in vitro* recording of PER2::LUC activity were done as described in Section 2.1.4, but tissue samples were exposed to recording medium containing different CORT concentrations. Corticosterone was dissolved in ethanol and later diluted in recording medium, aimed at final CORT concentrations of around 300 ng/ml (medium physiological concentration) and 900 ng/ml (high concentration). Due to ethanol volatility, the final concentrations were somewhat higher and ranged from 374.5 ng/ml to 556.5 ng/ml (medium-high) and 877 ng/ml to 1577.5 ng/ml (high), respectively. A pilot study was performed to assess if and how much the concentration of corticosterone added at the start of the recording would change over the 1 week recording period. Samples from the recording medium with and without liver tissue were collected at different time intervals and analyzed for CORT by radioimmunoassay (MP Biomedicals, Eschwege, Germany).

From each mouse, six liver samples were taken and cultured in separate dishes with pre-warmed recording medium with a high, medium or zero concentration of CORT (duplicates for each concentration). Coronal brain slices (200 μm) were prepared on a vibratome after which slices containing the SCN were selected and the SCN's from the left and right hemisphere were separated from each other and from the surrounding brain tissue using a scalpel. The left and right SCN samples were placed separately in dishes with culture plate inserts and pre-warmed recording medium. Because of the small size of the SCN and the limited number of sections containing this nucleus (1–2), SCN samples were only exposed to the zero and high concentration of CORT (if possible duplicates). For each mouse, the SCN from one hemisphere was placed in medium containing the high concentration of corticosterone, while the SCN from the other hemisphere served as control and was placed in medium without CORT.

#### 2.2.4 Data analysis

Period was analyzed as described in Section 2.1.5 and phase was determined by selecting the second peak of PER2::LUC rhythm. A Student's *t*-test was used to analyze the effects of corticosterone on period and phase of the PER2::LUC rhythm in the SCN and a one-way ANOVA with between-subjects factor CORT was used to test the effects on period and phase of the PER2::LUC expression rhythm in the liver. Effect sizes were calculated as previously described in Section 2.1.5.

## 3 Results

### 3.1 Experiment 1. Effects of social defeat stress on running-wheel activity and PER2 rhythms

#### 3.1.1 Circadian activity rhythm

Data from 5 animals were excluded from the analysis due to technical issues and loss of activity recordings, resulting in a total of 7 animals in the control group and 8 in the social defeated group. Also because of incomplete activity recordings, data from the Social Defeat block was analyzed until the 8th day.

Fig. 2A shows examples of activity recordings in a control and a social defeated mouse under constant dim red light; both displayed free-running periods slightly longer than 24 h. Overall, repeated measures ANOVA did not reveal any effect of defeat on free-running period (GROUP F_(1,13)_ = 3.00, p = 0.11, η^2^_p_ = 0.18; GROUP x TIME block interaction: F_(1,13)_ = 2.00, p = 0.20, η^2^_p_ = 0.12; Fig. 2B).

Fig. 2C depicts the mean circadian time of activity onset on the 10th baseline day and the 8th day of the Social Defeat block. Repeated measures ANOVA showed no difference between groups (F_(1,13)_ = 1.12, p = 0.31, η^2^_p_ = 0.08), neither a GROUP x DAY interaction (F_(1,13)_ = 0.73, p = 0.41, η^2^_p_ = 0.05).

**Fig. 3:**
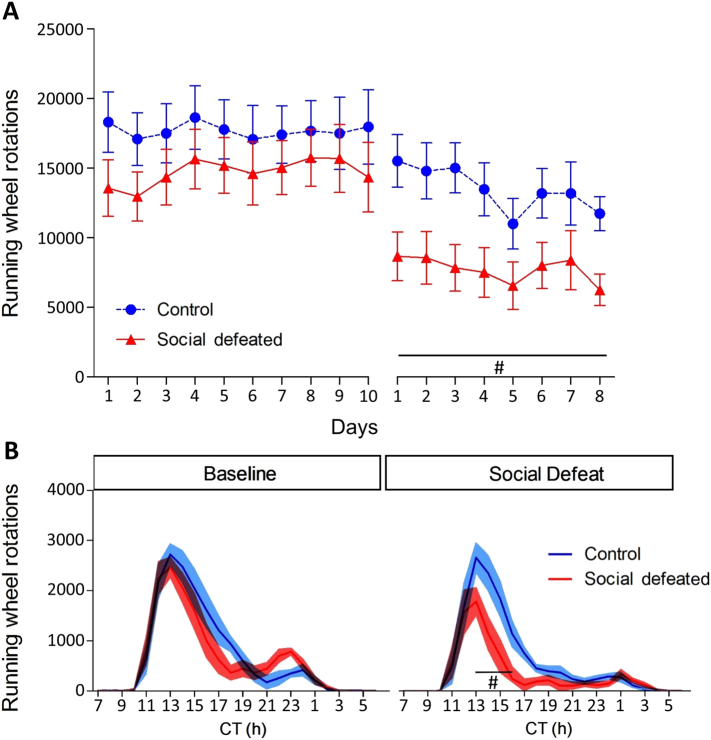
Panel A) Total running wheel rotations per day during Baseline and Social Defeat days. There was a difference between groups during Social Defeat. Symbols represent mean ± SEM. Panel B) Total running wheel activity per hour during Baseline and Social Defeat blocks. The Social defeated group ran less than the Control group between CT 13 and CT 16 during the Social Defeat block. Lines represent mean and colored area SEM. For both panels A and B, # indicates difference between groups.

The average number of running wheel rotations per day are represented in Fig. 3A. There was no difference in daily activity between groups during Baseline (F_(1,13)_ = 1.07, p = 0.32, η^2^_p_ = 0.08). During the Social Defeat block, defeated mice ran less than control animals (F_(1,13)_ = 7.95, p = 0.01, η^2^_p_ = 0.38). Repeated measures ANOVA also indicated an effect of DAYS (F_(7,91)_ = 2.19, p = 0.04, η^2^_p_ = 0.03); however, the post-hoc test did not detect differences among the days during Social Defeat.

Fig. 3B shows the average 24 h activity profiles of mice during the 10-day baseline block and during the experimental block. There was no difference between the groups during Baseline days. During the social defeat days there was a main effect of GROUP (F_(1,13)_ = 7.96, p = 0.01, η^2^_p_ = 0.38) and a GROUP x HOURS interaction (F_(23,299)_ = 2.63, p < 0.01, η^2^_p_ = 0.17). The *post-hoc* test indicated that Social defeated group ran less than Control group from CT 13 to CT 16.

#### 3.1.2 Circadian PER2::LUC rhythm

No data were obtained from a number of SCN and liver samples due to low expression of the PER2::LUC protein. In total PER2 expression data were obtained from SCN samples of 8 control and 8 socially defeated animals and from liver tissue of 8 control and 7 socially defeated animals.

**Fig. 4:**
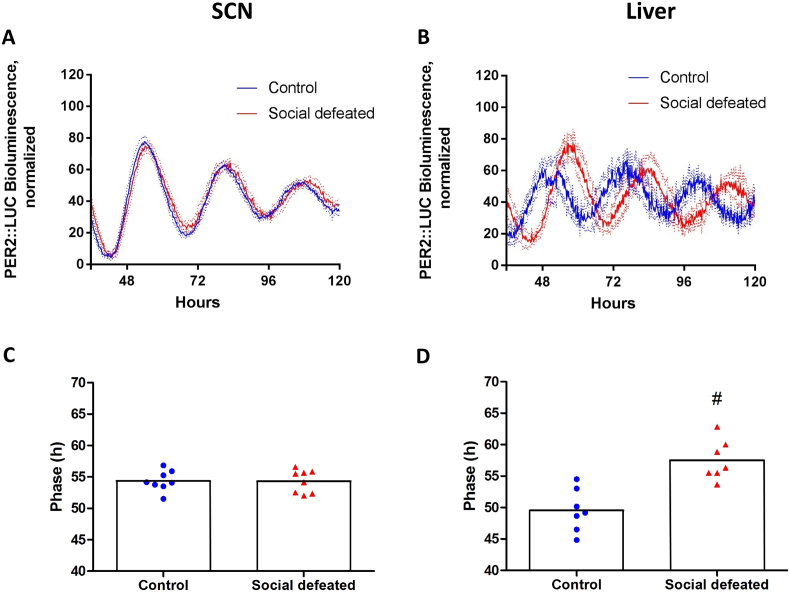
PER2::LUC rhythm in the SCN and Liver tissues collected from social defeated and control mice. Panels A and B) Normalized recording traces with subtracted baselines from SCN and liver slices, respectively. Lines represent mean and dotted lines SEM. Panels C and D) There was no difference between groups for the SCN, but phase was delayed for the Social defeated group in liver cultures. Bars represent mean and symbols represent each individual animal. In panel D, # indicates difference between groups.

Panels A and B from Fig. 4 show the averages of detrended normalized traces from bioluminescence rhythms in SCN and liver tissue, with the lowest and highest value in each sample trace corresponding to 0 and 100, respectively. Panels C and D show the average phase of the rhythm of PER2::LUC activity from the SCN and liver cultures. In the SCN samples, neither period nor phase were affected by prior social defeat stress (Student's *t*-test for period: t(14) = 0.42, p = 0.68, *d* = 0.21; for phase: t(14) = 0.06, p = 0.95, *d* = 0.03). For the liver samples, the test did not indicate a difference in period (t(13) = 0.42, p = 0.68, *d* = 0.22), but it did show a significant difference in the phase of the PER2 rhythm, which was delayed by about 8 h in the social defeated group (57.52 h ± 3.17) compared to the control group (49.44 h ± 3.16 h) (t(13) = 4.93, p < 0.01, *d* = 2.55).

### 3.2 Experiment 2. Direct effects of glucocorticoids on PER2 rhythm

Our pilot study showed that the CORT concentrations in the medium remained stable over a one-week recording period (Fig. S1). This was true for both the medium and high CORT concentrations.

Although CORT concentrations were stable across the 7-day recording period, the onset of CORT exposure differed, such that samples from half of the mice were collected and exposed to CORT starting at ZT 11 (towards the end of the normal resting phase of the mouse) and samples from the other half of the mice were collected and exposed to CORT starting at ZT 23 (towards the end of the normal active phase of the mouse).

**Fig. 5:**
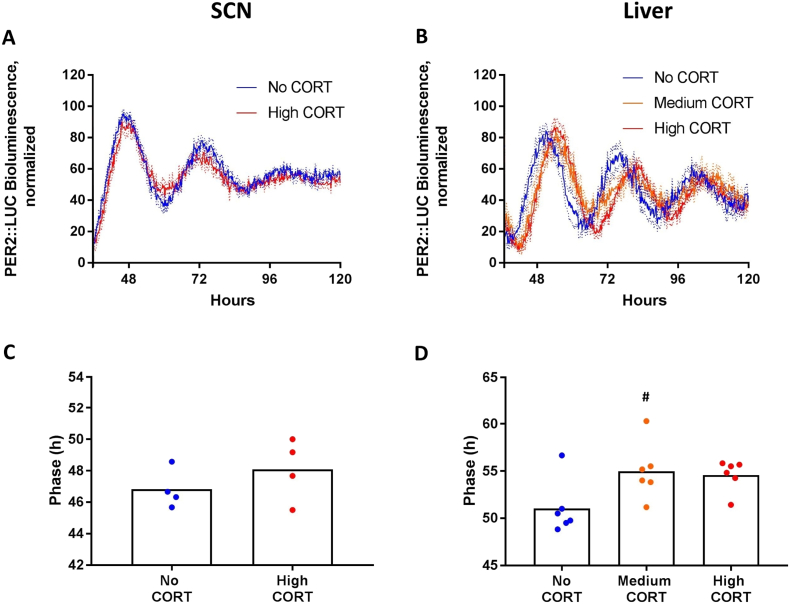
Effects of corticosterone on phase of PER2::LUC rhythm in tissues collected at ZT 11. Panels A and B) Normalized recording traces with subtracted baseline from SCN and liver tissues, respectively. Lines represent mean and dotted lines SEM. Panel C) Phase in the SCN. There was no effect of corticosterone. Panel D) Phase in the liver. Medium concentration of corticosterone delayed phase in the liver. Bars represent mean and symbols represent each individual animal. # Indicates difference from No CORT group.

**Fig. 6:**
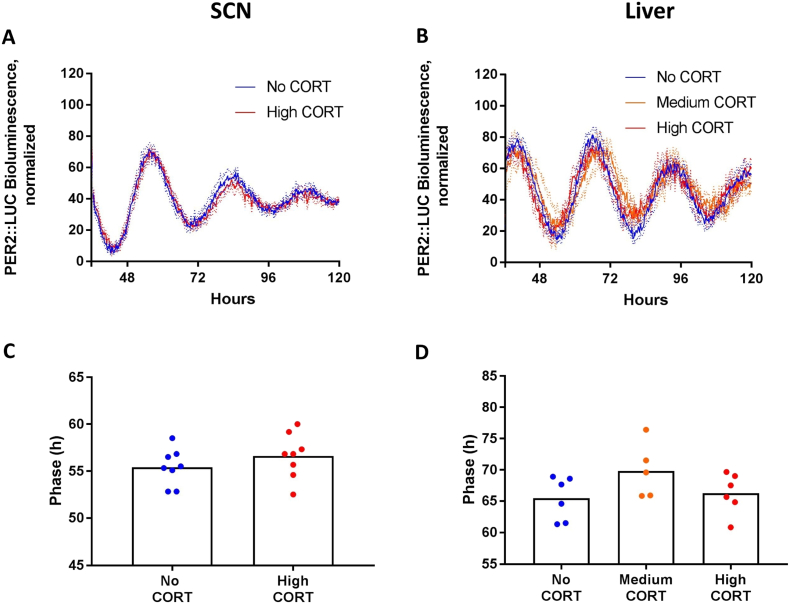
Effects of corticosterone on phase of PER2::LUC rhythm in tissues collected at ZT 23. Panels A and B) Normalized recording traces with subtracted baseline from SCN and liver tissues, respectively. Lines represent mean and dotted lines SEM. Panel C) Phase in the SCN. There was no effect of corticosterone on phase. Panel D) Period in the liver. ANOVA did not show effect of corticosterone on phase. Bars represent mean.

Not all tissue samples survived the preparation procedure and expressed sufficient levels of PER2. For samples collected at ZT 11 (Fig. 5), the data correspond to samples of SCN exposed to high CORT (4) or to no CORT (4) in the medium. A total of 6 successful measurements for each concentration were obtained for liver samples exposed to high, medium, or no CORT in the medium. For the samples collected at ZT 23 (Fig. 6), the data correspond to 8 samples of SCN exposed to high concentrations of CORT and 8 to no CORT. A total of 6, 5 and 6 successful measurements were achieved of liver samples exposed to high or medium CORT concentrations or no CORT, respectively.

Panels A and B of Fig. 5, Fig. 6 show normalized and averaged traces acquired from bioluminescence recordings of the SCN and liver tissues, respectively. Because of the dampening of the rhythm, data were analyzed from days 1 to 5. The amplitude variation of the rhythm varied among samples, and therefore this aspect was not analyzed.

Fig. 5 shows the effects of CORT at ZT 11 on the phase of PER2::LUC rhythm. Student's *t*-test did not reveal an effect of CORT on period (t(6) = 1.79, p = 0.12, *d* = 1.27) or phase (t(6) = 1.33, p = 0.23, *d* = 0.94) in the SCN. ANOVA did not show an effect of CORT on period (F_(2,15)_ = 0.06, p = 0.95, η^2^ < 0.01) but revealed an effect on phase (F_(2,15)_ = 4.25, p = 0.03, η^2^ = 0.36) in the liver. The post-hoc analysis showed a delayed phase of the second peak of the PER2::LUC rhythm in medium CORT concentration (54.99 h ± 1.23 h) compared to no CORT (51.04 h ± 1.17 h; p = 0.04), and a trend for effect of high concentration (54.58 h ± 0.68 h) compared to No CORT group (p = 0.07).

Fig. 6 illustrates the effects of corticosterone at ZT 23 on phase of PER2::LUC rhythm. There was no effect on period (t(14) = 0.015, p = 0.99, *d* < 0.01) or phase (t(14) = 1.09, p = 0.29, *d* = 0.5) in the SCN. ANOVA showed a trend for effect on period (F_(2,14)_ = 2.95, p = 0.085, η^2^ = 0.29) but no effect on phase (F_(2,14)_ = 1.78, p = 0.20, η^2^ = 0.20) in the liver.

## 4 Discussion

The present results confirm that daily social defeat stress in mice for 10 consecutive days strongly suppresses locomotor activity but does not affect the free-running period or phase of the activity rhythm under constant conditions (Ota et al., 2018). In agreement with the results for the activity rhythm was the finding that the rhythm of PER2 in the SCN collected after the stress period was unaffected by defeat. In contrast, the phase of the PER2 rhythm in liver tissue was significantly delayed (~8 h) in the defeated as compared to control mice. The results further showed that the phase of the PER2 rhythm in the liver was also delayed *in vitro* by direct application of CORT at ZT11, while the SCN tissue was, again, unaffected. Some effect size analyses showed large effects for non-significant results, but when the differences were statistically significant, the observed effect sizes were large. Together these findings support the hypothesis that stress can affect the peripheral liver oscillator but not the master clock in the SCN, possibly *via* a glucocorticoid mechanism.

Lesioning the SCN results in an arrhythmic activity pattern, indicating that this function is a direct output of the hypothalamic master clock (Richter, 1967; Stephan and Zucker, 1972). Both the activity data and the *in vitro* PER2 rhythm data thus indicate that the SCN is not disturbed by repeated stress. This finding is in agreement with previously published work in rodents subjected to a wide variety of different stressors (Richter, 1967; Meerlo et al., 2002). Our own previous work in rats had shown that social defeat stress either in the active phase or in the rest phase does not affect the phase and period of the free-running activity and temperature rhythms (Meerlo et al., 1997; Meerlo and Daan, 1998). More recently, we showed that even daily defeat stress in mice, during the active or resting phase, for 10-consecutive days had no effect on period or on phase of the locomotor activity rhythm under constant conditions (Ota et al., 2018).

Another recent study on the consequences of chronic intermittent social stress in mice reported small changes in the circadian period and phase of the activity rhythm, in apparent contrast to our current findings (Bartlang et al., 2015). Mice from two different strains (C57BL/6J and C57BL/6N) were subjected to the stress of a social conflict for 19 consecutive days, either in the light or in the dark phase, after which they were maintained in constant darkness to assess the free-running activity rhythms. The analysis suggested a stress-induced delay in the peak of the activity rhythm in both strains, especially when the animals were defeated in the dark phase. A small, but significant shortening of the free-running period by about 10 min was also reported only in the C57BL/6N mice (Bartlang et al., 2015). As discussed by the authors, the apparent phase delays might be explained by an altered rhythm shape rather than a true shift, perhaps as a result of conditioned fear-induced suppression of activity. Since we also observed activity suppression at certain circadian times, we opted to use the activity rise as a more robust phase marker, instead of the peak of activity, which might explain the different findings between the studies. In the experiments by Bartlang and colleagues, repeated defeats stress resulted in a small, albeit significant, 10-min shortening of the circadian period in the C57BL/6N strain. It is unclear why this result was strain-dependent, but the lack of a stress effect in the C57BL/6J mice is in line with the results from our own experiments that were performed in C57BL/6J mice (current results and Ota et al., 2018). Curiously, another study by the Bartlang group showed that the rhythm of PERIOD2 in the SCN was not affected by their protocol of chronic intermittent defeat stress (Bartlang et al., 2014). The lack of stress effect on the clock gene expression in the SCN is in agreement with the present study, showing no effect of repeated social defeat stress on the SCN PER2 rhythm *in vitro*. Together, these findings add to the general picture that the endogenous circadian pacemaker is highly resistant to stress.

Another model of stress-related disorders based on unpredictable mild chronic stress (UMCS) has produced findings that are similar to our results with social defeat stress. Logan and colleagues (2015) showed that mice exposed to UMCS also presented lower locomotor activity levels, and when PER2::LUC expression was analyzed in the SCN, there was a decrease in the amplitude, but nor phase or period changes after stress. Interestingly, when the authors used Quantitative Real-Time Reverse Transcriptase-Polymerase Chain Reaction, sampling 6 time points through a day and fitting the data to a harmonic regression, the expression of some clock genes appeared to show a phase shift. Although it is an appealing method to observe the expression of more genes, it is difficult to deduce whether there was a real effect on phase or period by fitting a curve through limited data points restricted to a single 24 h period, in comparison to multiple days of recording by means of the bioluminescence method.

In contrast to the master clock in the SCN, the liver responded to repeated social defeat stress. The phase of PER2::LUC rhythm was delayed in the liver tissue of defeated compared to control mice. Other studies have also reported phase shifts in clock gene expression in peripheral tissues, including a phase advance in the expression of this clock gene in the adrenal glands (Bartlang et al., 2014; Razzoli et al., 2014) and pituitary (Razzoli et al., 2014) of mice defeated during the light phase. Furthermore, restraint stress for 2 h/day for 3 days in the light phase causes phase advance in the expression of PER2::LUC protein in several tissues in mice, including the liver, whereas it has no effect in the SCN. The same effect is observed with mRNA expression of *Per1*, *Per2*, *Dbp*, and *Rev-erbα* in the kidney and *Per1* and *Per2* expression in the hippocampus and cortex (Tahara et al., 2015). The latter finding of stress-induced shifts in clock gene expression measured with different techniques *in vivo* and *in vitro* suggests these are true effects of stress and not a result of a different response to culturing of tissues for bioluminescence measurement (Leise et al., 2018).

The mechanism by which different stressors affect endogenous clocks in peripheral organs such as the liver, may involve multiple systems and pathways. Stress is a complex phenomenon associated with increased activity of a myriad of neuronal and neuroendocrine systems. Potential candidates for the stress effects on peripheral clocks are the hormones produced by the classical neuroendocrine stress systems, the Hypothalamic-Pituitary-Adrenal (HPA) axis and the Sympatho-Adrenal Medullary (SAM) system. In the present study, we specifically tested whether the liver clock is sensitive to glucocorticoids directly by exposing liver samples *in vitro* to CORT. We found a phase shift of the PER2 rhythm of liver tissue collected at ZT 11 and treated with the medium CORT concentration, similar to what we observed in liver tissue collected from animals that had been exposed to chronic intermittent social defeat stress.

Other studies with *in vitro* treatment have also reported direct effect of glucocorticoids on PER2 rhythm. In the nasal mucosa tissue of PER2::LUC mice, dexamethasone caused a maximum phase advance when administered at CT 18 and a maximum phase delay when administered at CT 12 (Honma et al., 2015). Embryonic fibroblasts from PER2::LUC knock-in mice treated with dexamethasone had an increase in PER2 protein levels and a phase delay of the gene expression rhythm, and when the cells were treated with a GR antagonist, these effects were blocked, showing that the glucocorticoid effect is dependent of this receptor (Cheon et al., 2013).

Our findings are also in agreement with a study by Tahara et al. (2015), who observed a phase advance of the peak of the PER2::LUC rhythm in peripheral tissues after exposure to the synthetic glucocorticoid dexamethasone, to norepinephrine or epinephrine, at ZT 4 for 3 consecutive days. Also, it was previously reported that dexamethasone injections can phase shift clock gene expression in liver, kidney, and heart tissue, but does not affect clock gene expression in the SCN (Balsalobre et al., 2000). In humans, treatment with Cortef (a synthetic hydrocortisone) for 6 days phase shifted PER2–3 and BMAL1 rhythm in peripheral blood mononuclear cells, but neither phase nor the amplitude of plasma melatonin rhythm were modified, indicating that the central clock was not affected by the glucocorticoid (Cuesta et al., 2015).

Similar to other studies using glucocorticoid treatments, and to our own results with social stress, we did not observe an effect of CORT on PER2 rhythm in the SCN tissue. Interestingly, a 6-week treatment of adrenalectomized mice with hydrocortisone in drinking water phase shifted PER1::LUC expression in different peripheral tissues, caused phase desynchrony in the liver and also advanced phase in the SCN (Pezük et al., 2012). The authors discussed that glucocorticoids could affect the master clock by disturbing the raphe nuclei, which sends inputs to the SCN. Although our previous studies did not indicate an effect of 10 days of social stress on the SCN, perhaps a more prolonged stress could have this effect in the living animal.

Interestingly, a recent study by Čečmanová et al. (2019) reported phase shifts of the PER2 rhythm in fetal SCN in response to dexamethasone, which was either a phase advance or phase delay depending on the time of the treatment. Importantly, it is known that at this early developmental phase, the SCN still contains glucocorticoid receptors, unlike the adult SCN (Rosenfeld et al., 1988). Therefore, this major component of the stress response is the most likely explanation for the discrepancy between the results obtained in fetal and adult SCN.

While the data from our *in vitro* corticosterone manipulation provide support for the hypothesis that effects of chronic social stress on the liver clock may be mediated by this hormone, they do not directly prove it. One limitation of our study is that our daily social stress protocol likely resulted in once-a-day peaks in corticosterone levels whereas the *in vitro* tissue cultures were exposed to near constant levels of corticosterone. In our experimental set-up it was not possible to mimic daily peaks in corticosterone *in vitro* without disturbing the tissues cultures and the ongoing bioluminescence measurements. Also, we do not exactly know the dynamics of the corticosterone levels in the 10-day chronic social defeat paradigm. In the current study we decided to not take repeated blood samples from the mice because that would have been an additional manipulation and potential stressor in itself (also in control animals). Importantly, while it is not excluded that the acute responses to the daily stressor may have diminished over time as a result of habituation, literature suggests that even after 10-days of social stress, rodents still display elevated levels of corticosterone than control mice (Macedo et al., 2018). Ultimately, proving an involvement of corticosterone in the effects of social defeat stress on the liver clock will require directly blocking corticosterone signaling *in vivo* during stress, for example, by adrenalectomy.

### 4.1 Conclusion

In conclusion, our experiments show that chronic social stress does not disturb the master clock in the SCN but it can phase shift the peripheral liver oscillator. Similar phase shifting effects of applying corticosterone directly on liver tissue suggest that the effects of stress may be mediated by adrenal glucocorticoids, but further studies are required to confirm this hypothesis.

The following is the supplementary data related to this article.

Fig. S1 Corticosterone concentration along the days. Corticosterone concentration in the medium of plates with and without liver tissue before closing the dish, within 1, 2, 4 and 7 days in the LumiCycle. Corticosterone levels seem to remain stable during the 7 days of recording. Fig. S1

## Declaration of competing interest

The authors declare no conflict of interest.
