# Supplementary material for: Social stress and glucocorticoids alter PERIOD2 rhythmicity in the liver, but not in the suprachiasmatic nucleus
Source: Horm Behav. 2020 Apr;120:104683. doi: 10.1016/j.yhbeh.2020.104683 (PMC7332991; doi:10.1016/j.yhbeh.2020.104683)
Supplement: Fig. S1 — Corticosterone concentration along the days. Corticosterone concentration in the medium of plates with and without liver tissue before closing the dish, within 1, 2, 4 and 7 days in the LumiCycle. Corticosterone levels seem to remain stable during the 7 days of recording. [file mmc1.docx]

Supplementary material

Figure S1. Corticosterone concentration along the days. Corticosterone concentration in the medium of plates with and without liver tissue before closing the dish, within 1, 2, 4 and 7 days in the LumiCycle. Corticosterone levels seem to remain stable during the 7 days of recording.

**
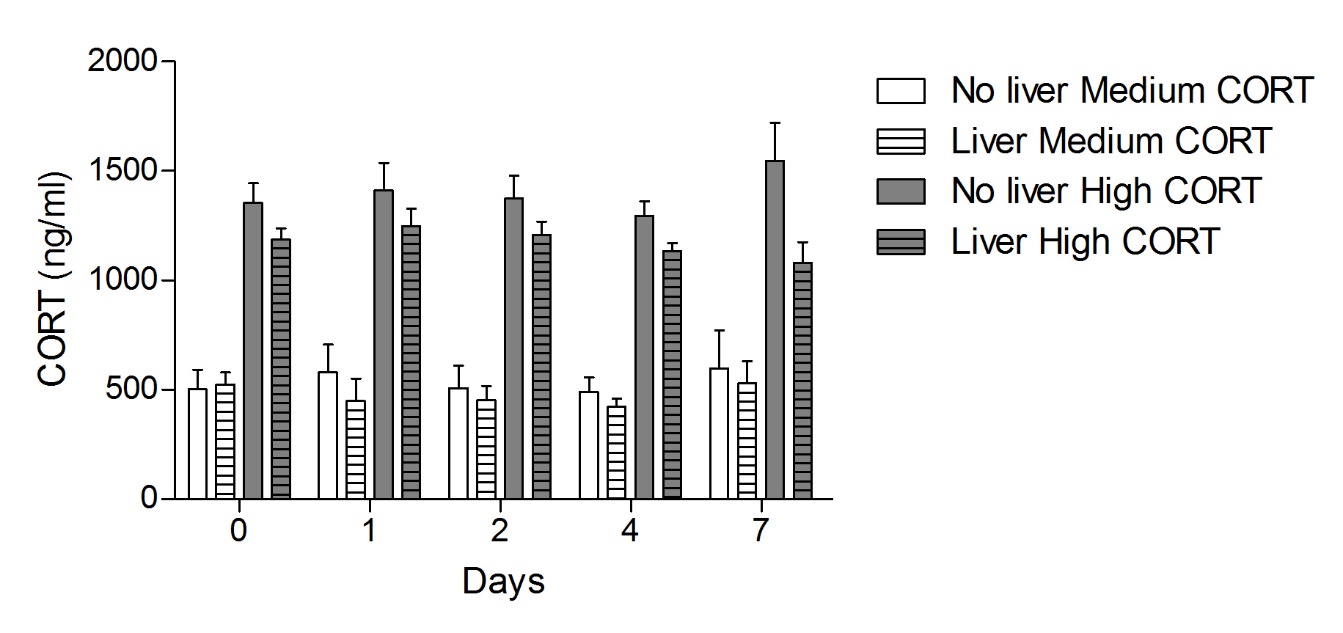
**

Figure S1.
